# Differential Gene Expression of *Porphyromonas gingivalis* in the Presence or Absence of Xanthohumol and Curcumin in a Dynamic In Vitro Biofilm Model

**DOI:** 10.3390/ijms262311315

**Published:** 2025-11-23

**Authors:** Enrique Bravo, Cristina Chamorro, David Herrera, Mariano Sanz

**Affiliations:** ETEP (Etiology and Therapy of Periodontal and Peri-Implant Diseases) Research Group, Faculty of Dentistry, Complutense University, 28040 Madrid, Spain; ebravofe@ucm.es (E.B.); cchamorro@ucm.es (C.C.); davidher@odon.ucm.es (D.H.)

**Keywords:** *Porphyromonas gingivalis*, oral biofilm, RNA sequencing, xanthohumol, curcumin, differential gene expression

## Abstract

This study aimed to characterize the transcriptional response of *Porphyromonas gingivalis* biofilms to treatment with xanthohumol and curcumin. A validated dynamic in vitro biofilm model, based on microbial growth under flow and shear conditions resembling the oral cavity, was used to develop mature biofilms of *P. gingivalis* on sterile ceramic calcium hydroxyapatite discs. Transcriptional profiles of biofilms, treated and untreated with both extracts, were obtained through RNA-Sequencing (RNA-Seq). The biofilm development and the lack of phenotypic effects from sublethal concentrations of xanthohumol and curcumin were confirmed via Scanning Electron Microscopy (SEM) and Confocal Laser Scanning Microscopy (CLSM). Reverse transcription quantitative PCR (RT-qPCR) was employed to verify differentially expressed genes identified by RNA-Seq. Xanthohumol and curcumin caused extensive reprogramming of *P. gingivalis* biofilm gene expression. Out of 1,973 genes, xanthohumol activated 173 and repressed 286, whereas curcumin activated 170 and repressed 163. These changes affected genes involved in membrane integrity, oxidative stress, transmembrane transport, and virulence, suggesting a mechanism of action that involves membrane disruption.

## 1. Introduction

Subgingival biofilms are collections of microorganisms closely associated and organized within an extracellular matrix, produced by the microbes themselves, which allows them to anchor to biotic or abiotic surfaces [1,2]. The various activities of these microbial communities, as well as the qualitative and quantitative changes in their composition, have been linked to the initiation and progression of periodontal diseases [3,4]. In fact, according to the 2018 Classification of Periodontal and Peri-implant Diseases and Conditions, dental biofilm-induced gingivitis and periodontitis are among the most important conditions listed [5,6].

One of the main contributors to the pathobiology of these conditions is *Porphyromonas gingivalis*, a Gram-negative anaerobic bacterium, considered a late colonizer in biofilm formation. Its pathogenicity is closely linked with its abundance within the biofilm, the expression of specific virulence factors, and its resistance to host inflammatory and immune responses [7,8,9,10,11,12]. In fact, the growth of *P. gingivalis* within the subgingival biofilm triggers the expression of a set of genes, mainly related to the cell wall, oxidative stress, virulence, transport, membrane proteins, and quorum sensing [13,14,15].

Although the primary approaches to controlling dental and subgingival biofilms are mechanically based, the use of chemical agents, whose mechanism of action is related to the inhibition of the previously described processes, represents a promising strategy for controlling the development and progression of periodontal and peri-implant diseases. Among these chemical agents, specific phytochemicals (natural substances of vegetal origin) have shown activity against recognized periodontal pathogens [16,17,18].

**Figure 1:**
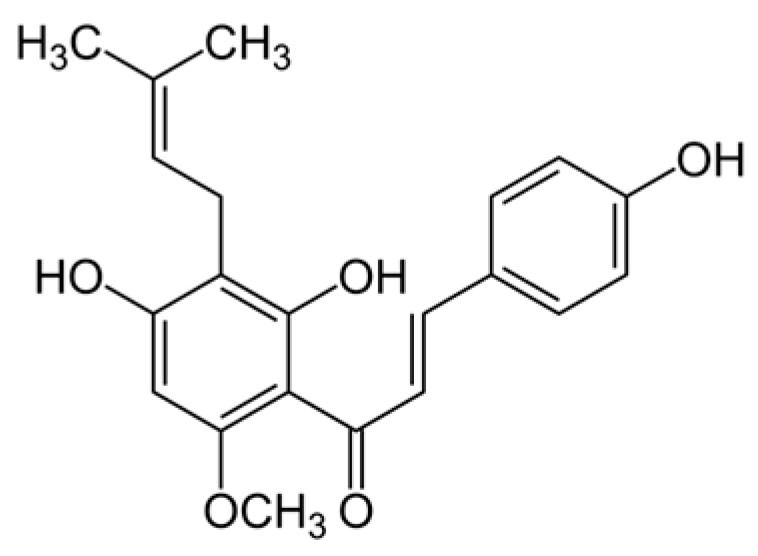
Chemical structure of xanthohumol.

A phytochemical of interest is xanthohumol (XN), a prenylated flavonoid (C_21_H_22_O_5_) from the female flowers of the hop plant (*Humulus lupulus*), which exhibits anti-inflammatory and antioxidant properties [19] (Figure 1). This extract has demonstrated a strong antimicrobial effect [20,21,22,23].

**Figure 2:**
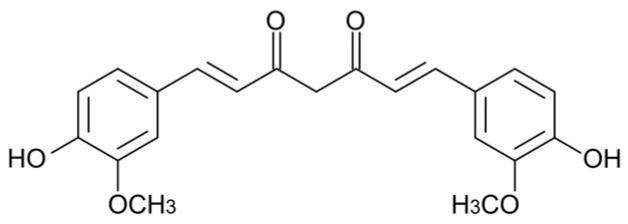
Chemical structure of curcumin.

A second promising phytochemical is curcumin (Cur), an aromatic polyphenol (C_21_H_20_O_6_) found in the rhizomes of turmeric (*Curcuma longa*) (Figure 2). This extract has antioxidant and anti-inflammatory properties [24,25,26], as well as bactericidal and fungicidal activity [27,28].

Recent research highlights the antimicrobial potential of XN and Cur against *P. gingivalis*. XN significantly reduces the viability and biomass of multispecies biofilms, developed on implant surfaces in vitro models, with an efficacy comparable to or better than chlorhexidine [29,30]. Cur also shows strong inhibitory effects on *P. gingivalis*: it disrupts key metabolic pathways and enzyme activities involved in bacterial growth [31], decreases adhesion and biofilm formation while downregulating major virulence genes [32], and inhibits gingipain activity, resulting in a notable reduction in biofilm [33]. Overall, these findings suggest that both compounds have antimicrobial potential for their use as adjuncts in the treatment of periodontitis. However, the mechanism of action remains unclear. Comparing the gene expression profiles of *P. gingivalis* biofilms with and without XN and Cur could reveal how both phytochemicals function as either antimicrobials or host response modulators. The complete genome characterization of *P. gingivalis* had been a significant milestone for gene expression studies of this bacterial species [34,35].

Therefore, this study aimed to characterize the differential gene expression of *P. gingivalis* in sessile growth with and without XN and Cur in a validated dynamic in vitro biofilm model.

## 2. Results

### 2.1. Minimum Inhibitory Concentrations of Xanthohumol and Curcumin Against P. gingivalis

The minimum inhibitory concentrations (MICs) of XN and Cur against *P. gingivalis* were established at 50 and 500 µM, respectively.

### 2.2. Scanning Electron Microscopy and Confocal Laser Scanning Microscopy to Monitor P. gingivalis Biofilm Development and the Effect of Treatments

**Figure 3:**
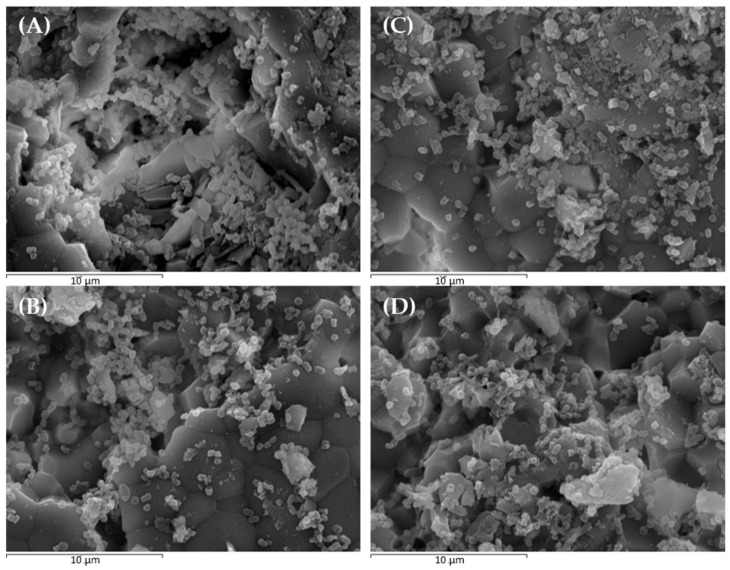
Scanning electron microscopy (SEM) images at 5000× magnification showing: (**A**) negative control biofilms incubated with phosphate-buffered saline; (**B**) negative control biofilms incubated with 0.25% dimethyl sulfoxide; (**C**) biofilms treated with 50 µM xanthohumol; and (**D**) biofilms treated with 500 µM curcumin (scale bar = 10 µm).

Scanning electron microscopy (SEM) images revealed the formation of a mature monospecies biofilm of *P. gingivalis*, where cocco-bacillary forms gathered in a highly organized three-dimensional structure (Figure 3A). Treatments with sublethal concentrations of dimethyl sulfoxide (DMSO), XN, and Cur, used in this study, did not alter the structure or organization of the developed biofilms (Figure 3B–D, respectively).

**Figure 4:**
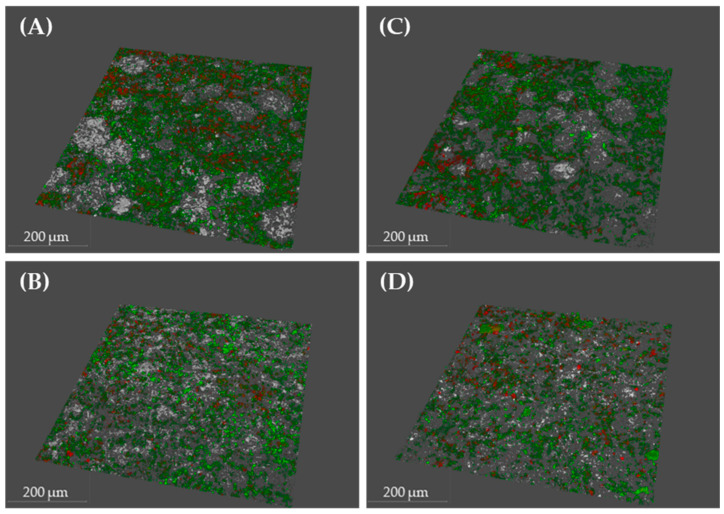
Confocal laser scanning microscopy (CLSM) images: (**A**) negative control biofilms incubated with phosphate-buffered saline (PBS); (**B**) negative control biofilms incubated with 0.25% dimethyl sulfoxide (DMSO); (**C**) biofilms treated with 50 µM xanthohumol (XN); and (**D**) biofilms treated with 500 µM curcumin (Cur). LIVE/DEAD^®^ BacLight Kit was used to stain live bacteria (green), dead bacteria (red), and disc surfaces (white). (scale bar = 200 µm).

Similarly, confocal laser scanning microscopy (CLSM) analysis showed no effect on bacterial density, cell viability, or biofilm roughness (Table 1 and Figure 4).

The minor differences among groups were not statistically significant.

### 2.3. Comparative Analysis of RNA-Sequencing-Obtained Transcriptomes

The obtained transcriptomes revealed 1973 genes with at least 10 counts in each experimental replicate, representing 91.55% of the total genes in the *P. gingivalis* ATCC 33277 strain [35]. The comparison between the transcriptional profiles after biofilm incubation in PBS and in 0.25% DMSO showed no gene regulation changes caused by the solvent at the selected concentration, with only one gene being overexpressed (Supplementary Table S1). Therefore, differential gene expression in the presence or absence of 50 µM XN and 500 µM Cur was evaluated by comparing the transcriptomes obtained after incubation with each extract against the profile obtained with 0.25% DMSO.

**Figure 5:**
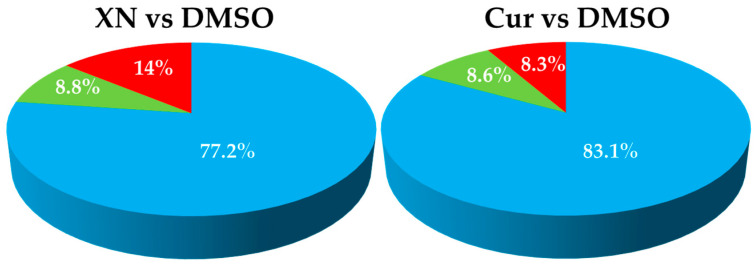
Proportion of upregulated and downregulated genes of *P. gingivalis* in response to exposure to xanthohumol (XN) and curcumin (Cur), compared to dimethyl sulfoxide (DMSO) as control.

As shown in Supplementary Table S1 and in Figure 5, XN induced the transcriptional activation of 173 genes and the repression of 286 (out of 1973 genes, representing 8.8% and 14%, respectively). Cur activated the expression of 170 genes and repressed the transcription of 163 (8.6% and 8.3%, respectively).

**Figure 6:**
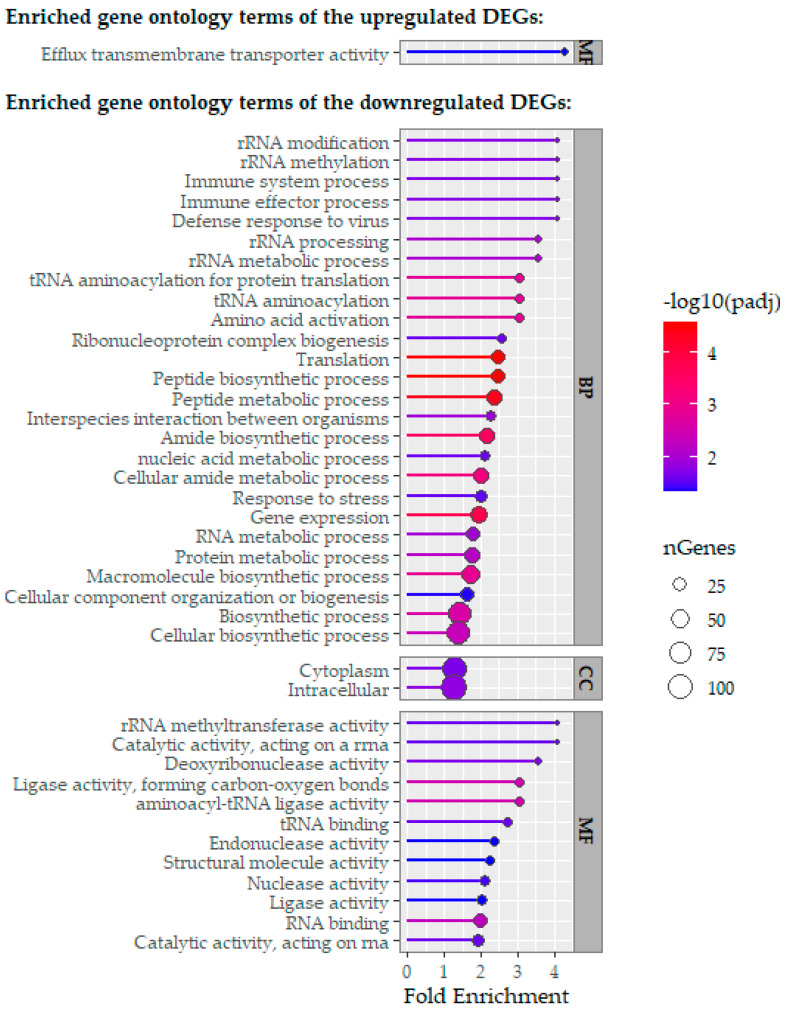
Functional categorization of significantly upregulated and downregulated genes of *P. gingivalis* in response to xanthohumol exposure, grouped by Gene Ontology (GO) categories: Biological Process (BP), Cellular Component (CC), and Molecular Function (MF). Enriched GO terms (*p*-value < 0.05) were identified using the hypergeometric test in ShinyGO after differential expression analysis with DESeq2.

**Figure 7:**
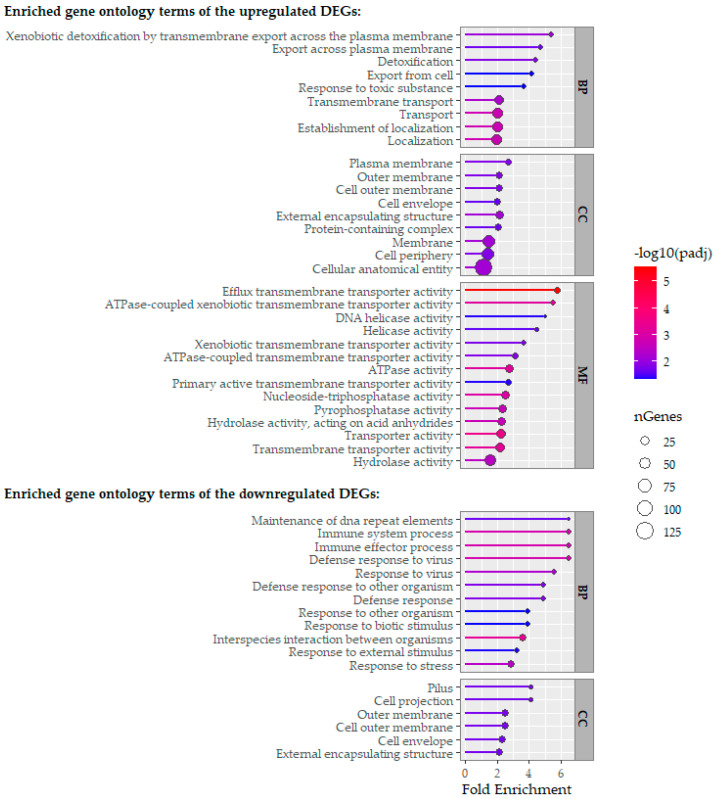
Functional categorization of significantly upregulated and downregulated genes of *P. gingivalis* in response to curcumin exposure, grouped according to Gene Ontology (GO) categories: Biological Process (BP), Cellular Component (CC), and Molecular Function (MF). Enriched GO terms (*p*-value < 0.05) were identified using the hypergeometric test implemented in ShinyGO, following differential expression analysis with DESeq2.

Differentially expressed genes across the tested conditions were classified into Gene Ontology (GO) categories: Biological Process (BP), Cellular Component (CC), and Molecular Function (MF) (Figure 6 and Figure 7).

Regarding the genes transcriptionally activated by Cur, transcripts involved in localization, transmembrane export, and response to toxic substances were overrepresented. Conversely, genes repressed by Cur treatment mainly related to stress response regulation and interactions with other microbial agents. For genes activated by XN, there was a strong enrichment in transport-related functions. In contrast, genes repressed by XN exposure showed significant enrichment in processes involving polypeptide and protein biosynthesis, intracellular translation, and RNA metabolism.

A total of 89 genes (4.5% of the *P. gingivalis* genome) were commonly upregulated by both extracts. Similarly, 140 genes (7.1%) were downregulaled under both conditions (Supplementary Table S1).

Principal Component Analysis (PCA) verified the quality and consistency of the data (Supplementary Figure S1). The samples are grouped according to the established experimental conditions, separated by the treatment variable, which confirms that the observed differences in gene expression are due to the experimental factor and not technical variability or noise. No outliers, batch effects, or technical errors were identified.

The estimated statistical power at n = 3 biological replicates per group was 0.75 for upregulated and 0.77 for downregulated genes in both the Cur vs. DMSO and XN vs. DMSO comparisons, further supporting the robustness of the differential expression results (Supplementary Figure S2).

To validate the sequencing results, the expression of twelve selected differentially expressed genes between target conditions was analyzed by reverse transcription quantitative PCR (RT-qPCR) (Supplementary Table S2). The differential gene expression patterns observed were consistent across both techniques.

## 3. Discussion

SEM and CLSM analyses confirmed the formation of a typical monotypic *P. gingivalis* biofilm with normal structure, complexity, and cell viability. Exposure to sublethal concentrations of XN and Cur used in this study did not affect any of these parameters (Figure 3 and Figure 4), thus ruling out artefacts that could bias the interpretation of the gene expression data.

The current study, based on RNA sequencing analysis, has demonstrated a significant impact of XN and Cur on the transcriptional profile of *P. gingivalis* after 60 s of exposure. This treatment duration matches the typical exposure time used during oral rinsing procedures or implant decontamination. Additionally, previous studies in which we showed the bactericidal effects of XN and Cur in subgingival biofilm models were conducted using this same treatment duration [29]. XN upregulated 8.8% and downregulated 14% of the genes, while Cur upregulated 8.6% and downregulated 8.3% (Figure 5).

XN exposure triggered the upregulation of genes related to transmembrane proteins, indicating a direct bacterial response to this compound. The increase in transcriptional expression of this gene cluster is consistent with the previously reported affinity of XN for specific bacterial membrane lipids, such as phosphatidylglycerol and cardiolipin (key components of the lipid bilayer), which in turn alters membrane integrity and function and induces oxidative stress through an increase in intracellular reactive oxygen species (ROS) [36]. This mechanism is consistent with the strong bactericidal effect of XN against *P. gingivalis*. Thus, these findings suggest a mechanism of action for XN involving membrane disturbance and oxidative stress in *P. gingivalis*. The ability of XN to destabilize the membrane structure of anaerobic bacteria and affect their viability by disrupting ion transport also supports this approach [22,37,38]. The notable downregulation of genes involved in polypeptide and protein biosynthesis, translation, and RNA metabolism in response to XN indicates a conserved bacterial stress response to phenolic compounds. This transcriptional pattern matches that seen in *Lactobacillus plantarum* after exposure to hydroxytyrosol, another phenolic compound that inhibits protein synthesis and RNA metabolism [39]. Furthermore, other polyphenols, such as ferulic and gallic acids, cause changes in Gram-negative bacterial membranes, promote ROS buildup, and inhibit vital biosynthetic pathways, including translation and RNA metabolism, thereby contributing to their bacteria-killing effects [40].

Like XN, Cur also activated genes related to transmembrane transport and detoxification, indicating an adaptive response by *P. gingivalis* aimed at maintaining cell viability under stress [30,31]. One proposed antibacterial mechanism of Cur is disrupting the plasma membrane and causing morphological changes due to cell wall alterations [31,41]. The activation of stress response genes coding for membrane- and wall-associated proteins supports this idea. Additionally, the upregulation of transmembrane transport genes may result from altered metabolism of several amino acids essential for *P. gingivalis* [31], which primarily uses amino acids rather than sugars as carbon sources derived from protein degradation [42]. This interpretation is reinforced by recent studies indicating that Cur disrupts bacterial membrane integrity and modulates membrane-associated processes, supporting the membrane as a central antimicrobial target [43,44,45]. Another suggested mechanism of Cur is inhibiting *P. gingivalis* growth by blocking dipeptidyl peptidase (DPP) activity [31]; however, the present study showed the opposite, with transcriptional activation of genes related to DPP7 and DPP III activity. Likewise, no inhibition of GTPase activity, reported in *Bacillus subtilis* [28], was observed here, possibly due to the low concentration of Cur used.

The present study also showed the inhibition of expression of genes related to gingipain production (*rgpA* and *porT*) and fimbriae formation (*fimA*, *fimC*, and *fimD*) in response to both Cur and XN. These gene groups are closely associated with *P. gingivalis* virulence. It has been suggested that *P. gingivalis* virulence can be reduced using Cur, due to its ability to inhibit the expression of gingipain and biofilm-related genes controlled by *quorum sensing* [28,33,46]. Downregulation of these same genes also occurs when *P. gingivalis* is exposed to higher concentrations of Cur or for longer incubation periods [32]. In the case of XN, the genes *vimE* and *vimF*, which are responsible for synthesizing the structural lipopolysaccharide, were also suppressed, potentially decreasing biofilm virulence.

Thus, both XN and Cur downregulate well-established *P. gingivalis* virulence genes and biofilm formation-related genes, although no downregulation of the luxS gene (quorum sensing system of *P. gingivalis* [41]) was detected. Comparatively, chlorhexidine (CHX), the reference antiseptic in managing periodontal diseases [42], shows similar action by targeting the bacterial cell membrane, where it electrostatically binds to membrane phosphates, increases permeability, and causes leakage of essential intracellular components [43]. This comparison is especially relevant, as CHX acts through physical membrane disruption, while XN and Cur regulate gene expression at the transcriptional level, targeting virulence factors (e.g., fimbriae, gingipains) and membrane integrity—effects not reported for CHX. This suggests that, beyond direct antimicrobial action, these compounds may reduce *P. gingivalis* pathogenicity, a critical factor in modulating periodontal inflammatory responses [44,45]. Additionally, XN and Cur exhibit greater cytocompatibility than CHX, maintain similar antimicrobial efficacy, and have the potential to lessen the cytotoxic effects of *P. gingivalis* outer membrane vesicles [30,40]. Therefore, amid the rising levels of antibiotic resistance [46], our findings highlight the potential of phytochemicals as alternative strategies for preventing and treating periodontal and peri-implant diseases.

This study has limitations that must be acknowledged for accurate interpretation of the results. The experimental conditions focused only on *P. gingivalis* in a biofilm-sessile state. It is important to note that the transcriptional profile of *P. gingivalis* varies significantly between sessile and planktonic growth [15]. Specifically, genes involved in transport, membrane proteins, and envelope components are overexpressed in biofilm growth, while stress response genes are often under expressed. Moreover, growth in complex polymicrobial communities, such as those found in the subgingival periodontal pocket, further alters gene expression, particularly regarding oxidative stress response, cell envelope structure, transposon activity, and metabolism [13]. These conditions were not simulated in this study, which was limited to a monospecies biofilm model. It should also be noted that sublethal concentrations of XN and Cur were used in this study to maintain enough cell viability for detecting induced transcriptional changes, which might otherwise be hidden under high mortality conditions. Therefore, the concentrations used here do not match those suggested for therapeutic purposes. In fact, previous research has shown that lethal concentrations of both compounds significantly reduce the microbial density in treated biofilms, leading to a strong bactericidal effect [29,30]. Finally, factors such as host immune response and patient physiological status, both of which can greatly influence *P. gingivalis* gene expression, were not considered in this in vitro biofilm model.

In conclusion, despite the considerations mentioned, transcriptomic analysis showed that both XN and Cur cause extensive reprogramming of *P. gingivalis* gene expression, impacting genes related to membrane integrity, oxidative stress, transmembrane transport, and virulence. These effects suggest a mechanism of action involving membrane disruption and a potential induction of oxidative stress, leading to transcriptional adaptive responses and suppression of key virulence factors. Therefore, both compounds emerge as promising therapeutic options for managing periodontal and peri-implant diseases.

## 4. Materials and Methods

### 4.1. Microbial Strains and Culture Conditions

The bacterial strain *Porphyromonas gingivalis* ATCC 33277 was used. It was cultured on blood agar plates (Blood Agar Oxoid No. 2; Oxoid, Basingstoke, UK), supplemented with 5% (*v*/*v*) sterile horse blood (Oxoid), 5.0 mg/L haemin (Sigma, St. Louis, MO, USA), and 1.0 mg/L menadione (Merck, Darmstadt, Germany) at 37 °C for 24–72 h under anaerobic conditions (10% H_2_, 10% CO_2_, and N_2_ balance).

Pure cultures of *P. gingivalis* were obtained during its exponential growth phase under anaerobic conditions in protein-enriched brain heart infusion (BHI) medium (Becton, Dickinson and Company, Franklin Lakes, NJ, USA). The medium was supplemented with 2.5 g/L mucin (Oxoid), 1.0 g/L yeast extract (Oxoid), 0.1 g/L cysteine (Sigma), 2.0 g/L sodium bicarbonate (Merck), 5.0 mg/L haemin (Sigma), 1.0 mg/L menadione (Merck), and 0.25% (*v*/*v*) glutamic acid (Sigma). Bacterial concentration was measured spectrophotometrically to develop a *P. gingivalis* suspension containing 10^8^ colony-forming units (CFU)/mL.

### 4.2. Minimum Inhibitory Concentrations of Xanthohumol and Curcumin Against P. gingivalis

To select the optimum concentration of XN and Cur, at which the growth of the strain of interest was not affected, MICs assays against *P. gingivalis* were conducted [47].

Isolated colonies of *P. gingivalis* were grown in a protein-enriched BHI-modified medium, adjusted to pH 7.2 (Becton, Dickinson and Company, Franklin Lakes, NJ, USA), which was supplemented with 2.5 g/L mucin (Oxoid), 1.0 g/L yeast extract (Oxoid), 0.1 g/L cysteine (Sigma), 2.0 g/L sodium bicarbonate (Merck), 5.0 mg/L haemin (Sigma), 1.0 mg/L menadione (Merck), and 0.25% (*v*/*v*) glutamic acid (Sigma), at 37 °C under anaerobic conditions (10% H_2_, 10% CO_2_ and N_2_ equilibrium).

The exponential growth phase was identified spectrophotometrically, with cultures consistently below an optical density (OD_550nm_) of 1.2. Once exponential growth was achieved, 200 μL of inoculum were transferred to a 24-well microplate, resulting in a final concentration of 10^6^ CFU/mL. Then, XN (NATECO^®^ GmbH & Co., Wolnzach, Germany) and Cur (Sigma-Aldrich^®^, Steinheim, Germany) were added at final concentrations of 6.25, 12.5, 25, 50, 100, 250, 500, and 1000 µM for both phytochemicals, using PBS as the negative control. These microplates were incubated for 24 h at 37 °C under anaerobic conditions. The minimum inhibitory concentrations (MICs) of each extract were determined on blood agar plates, on which 100 µL of each suspension was seeded. The plates were incubated for 72 h at 37 °C under anaerobic conditions. The lowest concentrations of XN and Cur that produced at least a 90% inhibition of bacterial growth were considered the MICs for *P. gingivalis*, with a lower concentration range selected to avoid effects on cell viability.

MIC assays were performed in triplicate, with appropriate controls for contamination.

### 4.3. In Vitro Dynamic Monospecies Biofilm Model

A validated dynamic biofilm in vitro model was used [48]. The system includes a sterile vessel where the modified BHI medium flows via a peristaltic pump. The bioreactor (Lambda Minifor© bioreactor, LAMBDA Laboratory Instruments, Sihlbruggstrasse, Switzerland) maintains the culture medium under the stable conditions of the oral cavity (temperature of 37 °C, pH of 7.2, and an anaerobic environment with 10% H_2_, 10% CO_2_, and N_2_ as the balance).

The vessel was inoculated with 5 mL of the previously described *P. gingivalis* suspension, containing 10^8^ CFU/mL, and incubated for 12 h under the specified conditions. When the culture reached the exponential phase, a second peristaltic pump was activated at a steady flow rate of 30 mL/h to initiate continuous culture and transfer the culture to the Robbins device, where sterile calcium hydroxyapatite (HA) ceramic discs [7 mm diameter (SD = 0.2) and 1.8 mm thick] (Clarkson Chromatography Products, Williamsport, PA, USA) were placed. The discs were maintained for 72 h under the same conditions inside the Robbins device until mature monospecies biofilms of *P. gingivalis* formed on the HA discs. Three Robbins devices were used sequentially to increase the sample size of each test [49].

### 4.4. Experimental Groups

After incubation for 72 h, the discs were removed from the Robbins device and treated in microplate wells for 60 s, containing 1 mL of XN (50 µM) and 1 mL of Cur (500 µM), both resuspended in 0.25% (*v*/*v*) DMSO (AppliChen GinbH, Darmstadt, Germany). One mL of PBS and 1 mL of 1% DMSO (*v*/*v*) were used as negative control (PBS) and to discard the possible impact of the solvent at the concentration used (DMSO).

Subsequently, the discs were washed sequentially in 2 mL of sterile PBS three times (immersion time per rinse, 10 s), to remove any unattached bacteria. Immediately after the treatments, samples were flash-frozen in liquid nitrogen to halt any transcriptional activity occurring after the intervention.

For each condition, the protocol was repeated over nine discs in six independent sets of experiments, thus analyzing a total of 54 discs (n = 54) for RNA sequencing (RNA-Seq) and one disc per set of experiment for CLSM and SEM (n = 6).

### 4.5. Scanning Electron Microscopy

Samples were extracted from the Robbins device, washed, treated with PBS, DMSO, XN, or Cur, and dried using increasing concentrations of ethanol. They were then coated with gold before analysis, following a previously standardized protocol by Blanc et al. [48]. The samples were examined using a JSM 6400 electron microscope (JSM6400, JEOL, Tokyo, Japan).

This analysis was performed at the National Centre of Electron Microscopy (*In-stalación Científico-Técnico Singular; ICTS*) at the Moncloa Campus of the Complutense University of Madrid (Madrid, Spain).

### 4.6. Confocal Laser Scanning Microscopy

Samples were extracted, washed, treated with PBS, DMSO, XN, or Cur, and stained using the LIVE/DEAD^®^ BacLight^TM^ bacterial viability kit solution, following the protocol described by Blanc et al. [48].

A Leica^®^ LCS SP8 STED 3X inverted microscope (Mannheim, Germany) was used to capture non-invasive confocal images of the formed biofilms. COMSTAT 2.1 software [www.comstat.dk (accessed on February and March 2025)] was employed to calculate the biofilm biomass in cubic micrometres per square micrometre (µm^3^/µm^2^) and the roughness coefficient (Ra*) from the CLSM data. The analysis was conducted at the Margarita Salas Biological Research Centre (*Centro de Investigaciones Biológicas, Consejo Superior de Investigaciones Científicas*—CIB-CSIC), located on the Moncloa Campus of the Universidad Complutense de Madrid (Madrid, Spain).

### 4.7. Total RNA Isolation

After the four treatments (PBS, DMSO, XN, and Cur), the discs were rinsed in 2 mL of sterile PBS three times for 10 s each to remove cells not attached to the biofilm. They were then vortexed in 1 mL of PBS for 2 min at full power to disaggregate the biofilm, followed by centrifugation at 13,000 rpm for 3 min at room temperature to obtain sample pellets. Eighteen pellets from each replicate of each treatment (PBS, DMSO, XN, and Cur), obtained from two different experimental sets, were sequentially and cumulatively resuspended in 1 mL of sterile PBS. This process represented each biological replicate for each condition.

The RNeasy Protect Bacteria Kit (Qiagen, Hilden, Germany) was used to extract total RNA from the samples, following the manufacturer’s instructions. The quantity and quality of the RNA obtained were evaluated using the Agilent 2100 Bioanalyzer (Agilent Technologies, CA, USA). All samples used in this study had an A_260nm_/A_280nm_ ratio of at least 2.0.

### 4.8. RNA Sequencing

DNA libraries were prepared from the depleted RNA using the NEBNEXT Ultradirectional RNA Library Prep Kit (New England Biolabs, MA, USA), following the manufacturer’s instructions. In brief, the depleted RNA was fragmented and converted into double-stranded cDNA. Illumina sequencing adapters were then attached to the cDNAs, and the library was enriched through limited polymerase chain reaction (PCR). DNA fragments with 300–450 bp inserts were then sequenced in parallel at the Genomics Unit (Complutense University of Madrid).

Raw sequencing reads were initially evaluated with FastQC (version 0.12.1) [1] to analyze quality metrics such as base quality scores, duplication levels, and GC content. Then, filtering and trimming were carried out with AfterQC (version 0.9.6) [50], which automatically removed low-quality reads, trimmed adapters, and corrected sequencing errors.

High-quality reads were aligned to the *Porphyromonas gingivalis* ATCC 33277 reference genome (GenBank accession: GCA_000010505.1, ASM1050v1) using Bowtie2 (version 2.5.2) [51]. The genome was indexed with the bowtie2-build algorithm. Paired-end reads were aligned in global mode, and only those mapping concordantly and uniquely were kept for downstream analysis. Alignment files in SAM format were converted to compressed BAM format using SAMtools (version 1.17) [52], and the alignments were visualized with the Integrative Genomics Viewer (IGV) [53].

To estimate rRNA contamination, reads were aligned to rRNA databases using SortMeRNA (version 4.3.6) [54], and the proportion of rRNA-derived reads was then quantified.

Gene-level quantification was carried out using featureCounts [55] from the Subread package (version 2.0.5), assigning reads to genomic features based on the annotation file linked to the reference genome. Only fragments that mapped concordantly and exactly once were counted. The resulting count matrix was then used for differential gene expression analysis.

The gene count matrix was imported into R (version 4.3.3) for differential expression analysis using DESeq2 (version 1.42.1) [56]. Genes with more than 10 reads in at least three samples were retained for downstream analysis, resulting in a filtered dataset of 1973 genes (91.55% of the original gene set). This filtering step discarded 182 genes (8.45%) due to low or absent expression. Count data were normalized using the median-of-ratios method, and a negative binomial generalized linear model was fitted. Wald tests were used to assess differential expression, with *p*-values adjusted via the Benjamini–Hochberg method. Genes with an adjusted *p*-value < 0.05 and a log_2_ fold change (LFC) > 0.585 (upregulated) or <−0.60 (downregulated) were considered significantly differentially expressed.

Exploratory analysis was conducted using principal component analysis (PCA) to examine sample grouping by experimental condition and assess the consistency of replicates.

To gain insights into the biological functions linked to the differentially expressed genes, Gene Ontology (GO) enrichment analysis was conducted using the web-based tool ShinyGO (version 0.81) [http://bioinformatics.sdstate.edu/go/ (accessed on January 2025)]. This analysis categorized regulated genes into three GO groups: Biological Process (BP), Cellular Component (CC), and Molecular Function (MF), identifying significantly overrepresented functional terms (*p* < 0.05).

Power calculations were performed to evaluate the ability of the RNA-Seq experimental design to detect differentially expressed genes under each treatment condition (Curcumin vs. DMSO and Xanthohumol vs. DMSO). Analyses were based on empirical mean expression levels and dispersion estimates from the DESeq2 model, and genes were classified as upregulated (log_2_ fold change > 0.585) or downregulated (log_2_ fold change < −0.6) using the same thresholds applied in the DESeq2 differential expression analysis. For each comparison, power was estimated as a function of sample size (2–8 replicates per group) using the RNASeqPower package (version 1.42.0), with an alpha level of 0.05 and the global coefficient of variation derived from normalized counts. Power curves were computed separately for up- and downregulated genes to account for potential asymmetries in expression magnitude or dispersion, providing a realistic assessment of the experimental design.

### 4.9. Reverse Transcriptase Quantitative Polymerase Chain Reaction Validation

To confirm the differential expression results by RT-qPCR, twelve genes were chosen (three overexpressed and three repressed with XN, and three overexpressed and three repressed with Cur, always compared to the profile obtained with the DMSO control).

The cDNA was synthesized from 1 μg of total RNA using the PrimeScript^TM^ RT Reagent Kit (Takara, Kusatsu, Japan), according to the manufacturer’s instructions.

Specific primers were designed using the Universal Probe Library Roche (Roche Diagnostics) software tool (https://www.sigmaaldrich.com/JP/ja/product/roche/upl71thru80?srsltid=AfmBOor-3e0nXRaA3AMLJ3-IbesGZZOUgYZDOyJWuEwop6EtJ7LBJzWR (accessed on 21 October 2025)) (Supplementary Table S3). Primers and probes were supplied by Life Technologies Invitrogen (Carlsbad, CA, USA), Applied Biosystems (Carlsbad, CA, USA), and Roche (Roche Diagnostic GmbH, Mannheim, Germany). All quantifications were normalized to the 16S rRNA gene of *P. gingivalis*.

The qPCR reaction was performed using 1 μL of each cDNA per well and 9 μL of a mixture containing 10 μM of each primer, 5 μL of Fast Enzyme ExTaq (SYBR Green) (Takara, Kusatsu, Japan), and nuclease-free water (Roche) to reach a final volume of 10 μL on LightCycler 480 Multiwell-384 optical plates (Roche). PCR were carried out on a LightCycler^®^ 480 II thermal cycler (Roche Diagnostic GmbH, Mannheim, Germany). The amplification programme included an initial cycle at 95 °C for 3 min, followed by 40 cycles of 95 °C for 10 s, 60 °C for 20 s, and 72 °C for 1 s, and one final cycle at 95 °C for 5 s, 65 °C for 1 min, and 97 °C. The results were analyzed using the comparative Ct method (ΔΔCt) [56]. Each gene was tested in triplicate and compared with the results obtained from RNA-Seq analysis.

### 4.10. Statistical Analysis

Bacterial biomass reported after analysis by CLSM was expressed in µm^3^/µm^2^. Data are shown as means and SDs, and the Shapiro–Wilk goodness-of-fit test was used to assess data normality. When the two datasets compared showed a normal distribution, a T-test with Welch’s correction was applied. Statistically significant differences were considered for *p*-values <0.05. Present data were analyzed with the GraphPad Prism version 8.0.1 software.

## Figures and Tables

**Table 1:** Bacterial density, viability, percentage and roughness coefficient (Ra*) obtained by quantification of CLSM images.

Condition	Bacterial Density (µm^3^/µm^2^)		% Viability		Roughness Coefficient (Ra*)	
	Mean	(SD)	Mean	(SD)	Mean	(SD)
PBS	8.4	1.64	79.29	4.79	0.61	0.15
DMSO	10.33	1.24	78.57	3.1	0.51	0.06
XN	10.61	2.13	72.59	12.32	0.49	0.15
Cur	8.23	0.57	78.24	5.95	0.63	0.09

## Data Availability

All data are available within the article and its Supplementary Materials.

## Supplementary Materials

The following supporting information can be downloaded at: https://www.mdpi.com/article/10.3390/ijms262311315/s1.
