# Supplementary material for: Differential Gene Expression of Porphyromonas gingivalis in the Presence or Absence of Xanthohumol and Curcumin in a Dynamic In Vitro Biofilm Model
Source: Int J Mol Sci. 2025 Nov 23;26(23):11315. doi: 10.3390/ijms262311315 (PMC12691774; doi:10.3390/ijms262311315)

Supplementary Figure S1. Principal Component Analysis (PCA) graphs. Each point represents an RNA-Sequencing (RNAseq) sample, and the percentage on each axis indicates the proportion of variance explained by each principal component (**PC1, Principal Component 1; PC2, Principal Component 2**).

The **left panel** shows the arrangement of the cases [xanthohumol (XN) and curcumin (Cur)] and controls [phosphate buffer saline (PBS) and dimethyl sulfoxide (DMSO)] on opposite sides of PC1 (X-axis, 70 % variance) indicates the existence of a statistically significant number of differentially expressed genes between the two groups. The second dimension (PC2, Y-axis) is orthogonal to the first, constitutes the second source of variation, and has a much smaller effect on the differential expression of genes, as it only explains 13% of the variation observed in the data. This component separates the samples treated with XN, which are located at negative Y-axis values, from those treated with Cur, which are located at positive Y-axis values. The controls are located at values close to zero.

The **right panel** shows the same PCA colored by experimental replicate (_1, _2, _3), corresponding to three independent experiments conducted under the same conditions. No batch effect was observed, as samples grouped by treatment condition rather than by experimental replicate, confirming that the variance reflects biological rather than technical differences.

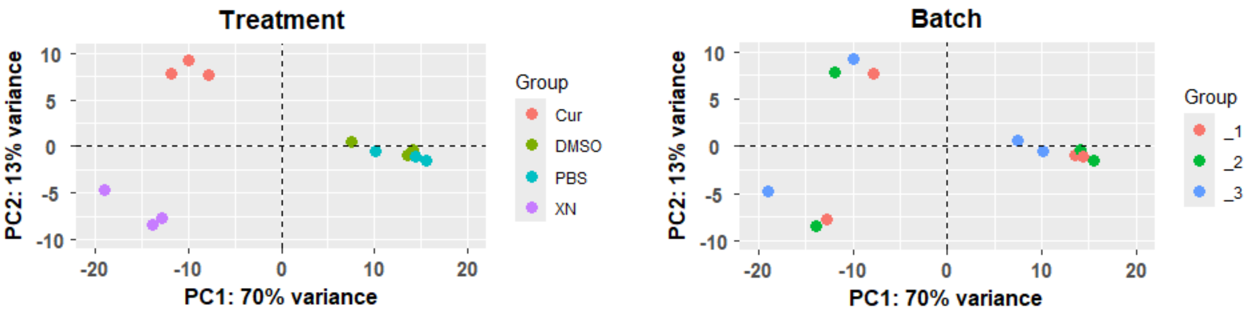

Supplement: Supplementary file 1 [file ijms-26-11315-s001.zip › Supplementary Figure S1.pdf]
