# Supplementary material for: Differential Gene Expression of Porphyromonas gingivalis in the Presence or Absence of Xanthohumol and Curcumin in a Dynamic In Vitro Biofilm Model
Source: Int J Mol Sci. 2025 Nov 23;26(23):11315. doi: 10.3390/ijms262311315 (PMC12691774; doi:10.3390/ijms262311315)

Supplementary Figure S2. Statistical power analysis for detection of up- and down-regulated genes *of P. gingivalis* in response to xanthohumol (XN) and curcumin (Cur) treatments compared to dimethyl sulfoxide (DMSO) as control.

Power curves show the probability of detecting differentially expressed genes as a function of replicate number per group. Solid lines represent up- (blue) and down-regulated (red) genes; the dashed vertical line indicates n = 3 replicates used in this study.

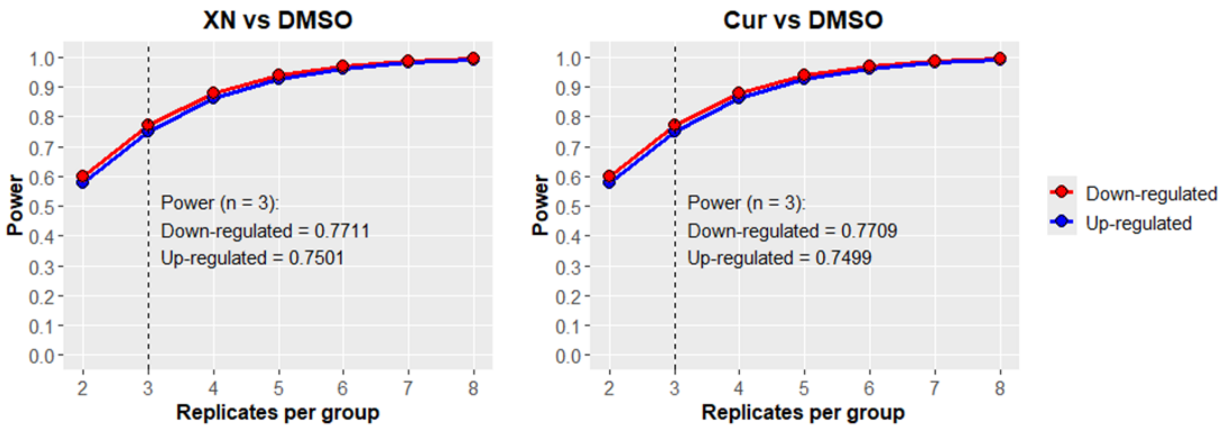

Supplement: Supplementary file 1 [file ijms-26-11315-s001.zip › Supplementary Figure S2.pdf]
