# Supplementary material for: Differential Gene Expression of Porphyromonas gingivalis in the Presence or Absence of Xanthohumol and Curcumin in a Dynamic In Vitro Biofilm Model
Source: Int J Mol Sci. 2025 Nov 23;26(23):11315. doi: 10.3390/ijms262311315 (PMC12691774; doi:10.3390/ijms262311315)
Supplement: Supplementary file 1 [file ijms-26-11315-s001.zip › Supplementary Table S2.pdf]

Supplementary Table S2. Differential gene expression between xanthohumol (XN) and dimethyl sulfoxide (DMSO), and between curcumin (Cur) and DMSO, obtained by RNA-Sequencing (RNA-Seq) and expressed as log2 gene expression fold-change (the ratio of gene expression in one experimental condition relative to another), and by reverse transcription quantitative polymerase chain reaction (RT-qPCR), expressed as means and standard deviations (SDs).

Comparison	Gen ID	log2 fold change		
		RNA-Seq	RT-qPCR	
			Mean	SD
XN vs DMSO	PGN_0448	4.89	2.63	1.13
XN vs DMSO	PGN_0450	5.70	2.54	1.15
XN vs DMSO	PGN_1208	2.65	3.76	1.81
XN vs DMSO	PGN_0178	0.17	0.10	0.02
XN vs DMSO	PGN_0680	0.28	0.09	0.02
XN vs DMSO	PGN_1648	0.26	0.17	0.12
Cur vs DMSO	PGN_1309	3.40	2.12	0.70
Cur vs DMSO	PGN_1343	6.32	3.22	0.48
Cur vs DMSO	PGN_1347	3.89	2.23	0.17
Cur vs DMSO	PGN_0348	0.35	0.34	0.10
Cur vs DMSO	PGN_1964	0.31	0.32	0.09
Cur vs DMSO	PGN_1965	0.30	0.29	0.13
