# Supplementary material for: Differential Gene Expression of Porphyromonas gingivalis in the Presence or Absence of Xanthohumol and Curcumin in a Dynamic In Vitro Biofilm Model
Source: Int J Mol Sci. 2025 Nov 23;26(23):11315. doi: 10.3390/ijms262311315 (PMC12691774; doi:10.3390/ijms262311315)
Supplement: Supplementary file 1 [file ijms-26-11315-s001.zip › Supplementary Table S3.pdf]

Supplementary Table S3. Primers used for Reverse Transcription-quantitative PolymeraseChain Reaction (RT-qPCR).

Locus name	Putative identification	Primer Sequence	
PGN_0450	Putative RNA polymerase	Forward 5′→ 3′	ATGTGCCGGACGAAGATCTC
		Reverse 5′→ 3′	AGCGAATCCAATGCTTGCTG
PGN_0448	ATP-binding protein	Forward 5′→ 3′	CAGTGGAGAACGTTGCCCTA
		Reverse 5′→ 3′	TGGCCATCTCATTGCGTTTG
PGN_1208	Chaperone ClpB	Forward 5′→ 3′	CGTCTGCGTATGGAAGTGGA
		Reverse 5′→ 3′	CAAGTTGCTTGATCCTGCGC
PGN_1648	Putative 50S ribosomal protein	Forward 5′→ 3′	GTAGAGAGTGGTGCAAGCGT
		Reverse 5′→ 3′	AAGGAGAACCCACGGCGATA
PGN_0680	Signal peptide peptidase	Forward 5′→ 3′	TCTGGACAACCTTTCCGTCG
		Reverse 5′→ 3′	TCTTGAAATCCTGCAACGCG
PGN_0178	DUF3575 domain-containing protein	Forward 5′→ 3′	ATACGCCATTGGTGCAGGTC
		Reverse 5′→ 3′	GCTGATCGATGCCTCCAAGT
PGN_1343	ATP-binding protein	Forward 5′→ 3′	TGTCATCGCATTGGCACTCT
		Reverse 5′→ 3′	TCGGAATATCCAGCCCGTTG
PGN_1347	Beta-barrel family protein	Forward 5′→ 3′	ATTCAGGTTCCATCACGGCC
		Reverse 5′→ 3′	GGACTTGGCCGTCAGAAAGA
PGN_1309	Ferrous iron transport protein B	Forward 5′→ 3′	CAAATCGGTGGAGTCGGGAT
		Reverse 5′→ 3′	TTGAGTCCCATCAGCCAACC
PGN_1964	Helicase Cas3 core	Forward 5′→ 3′	CGGAAAGAGACCCAAGCCAT
		Reverse 5′→ 3′	TGTCCCAATGTGGATCCTGC
PGN_0348	Universal stress protein	Forward 5′→ 3′	CGCGAGAAGAACTTCCCGAA
		Reverse 5′→ 3′	ATCACATCTTCCGGAGCACC
PGN_1965	Hypotetical protein	Forward 5′→ 3′	GTGATGCTACACCCGTCTCT
		Reverse 5′→ 3′	CAAAGATGCTGTCGAGCTGC
